# The chromosomal passenger complex and the spindle assembly checkpoint: kinetochore-microtubule error correction and beyond

**DOI:** 10.1186/1747-1028-3-10

**Published:** 2008-05-28

**Authors:** Gerben Vader, André F Maia, Susanne MA Lens

**Affiliations:** 1 Laboratory of Experimental Oncology, Department of Medical Oncology, University Medical Center Utrecht, The Netherlands; 2 Institute for Molecular and Cell Biology, Porto, Portugal; 3 Whitehead Institute for Biomedical Research, Cambridge, Massachusetts, USA

## Abstract

During mitosis, correct bipolar chromosome attachment to the mitotic spindle is an essential prerequisite for the equal segregation of chromosomes. The spindle assembly checkpoint can prevent chromosome segregation as long as not all chromosome pairs have obtained bipolar attachment to the spindle. The chromosomal passenger complex plays a crucial role during chromosome alignment by correcting faulty chromosome-spindle interactions (*e.g*. attachments that do not generate tension). In the process of doing so, the chromosomal passenger complex generates unattached chromosomes, a specific situation that is known to promote checkpoint activity. However, several studies have implicated an additional, more direct role for the chromosomal passenger complex in enforcing the mitotic arrest imposed by the spindle assembly checkpoint. In this review, we discuss the different roles played by the chromosomal passenger complex in ensuring proper mitotic checkpoint function. Additionally, we discuss the possibility that besides monitoring the presence of unattached kinetochores, the spindle assembly checkpoint may also be capable of responding to chromosome-microtubule interactions that do not generate tension and we propose experimental set-ups to study this.

## Background

After the initial description of the striking and dynamic localisation of the chromosomal passenger proteins [1], the function of these proteins during mitosis has been intimately linked with their localisation at specific structures in the mitotic cell. Whereas various proteins show a similar localisation pattern (*e.g*. TD60, Plk1) [2,3], we refer to the chromosomal passenger complex as the complex consisting of Aurora B kinase, Inner Centromere Protein (INCENP), borealin and survivin. Within the chromosomal passenger complex Aurora B is the enzymatic core that is activated and guided to its specific locations in the mitotic cell by INCENP, borealin and survivin [4,5].

In prophase, the chromosomal passenger complex localises to the chromosome arms, where it controls mitotic chromosome structure and organisation. Concentration at the centromeres during prometaphase reflects its essential function in between the paired kinetochores (*i.e*. large multiprotein complexes that assemble on the centromeres of sister-chromatids constituting their microtubule binding sites) to control and regulate proper kinetochore-microtubule interactions. Relocalisation of the chromosomal passenger complex to the central spindle and the equatorial cell cortex during anaphase and to the midbody in telophase, is essential for the proper function of the contractile ring and for final abcission, collectively ensuring cytoplasmic division [6].

Evidently, proper localisation of the chromosomal passenger complex at the right time is essential for faithful execution of mitosis [4]. In this review we summarize and discuss the current understanding of chromosomal passenger complex function in (pro)metaphase when it is localised at the inner centromere, with a specific focus on how this protein complex influences the control mechanism that monitors chromosome alignment, the spindle assembly checkpoint (also known as the spindle checkpoint).

### The spindle assembly checkpoint

The spindle assembly checkpoint guards the metaphase to anaphase transition by monitoring the presence of unattached and improperly kinetochores. It inhibits the anaphase promoting complex/cyclosome (APC/C), a multisubunit E3-ubiquitin ligase that targets at least two essential mitotic regulators, securin and cyclin B, for proteasomal destruction. The APC/C functions in conjunction with two different specificity factors, Cdc20 or Cdh1 of which Cdc20 is essential for destruction of securin and cyclin B in metaphase and hence for the onset of anaphase and mitotic exit [7]. Cdc20 appears to be the primary target of the spindle assembly checkpoint as it is found in an inhibitory complex with the checkpoint proteins Mad2, Mad3/BubR1 and Bub3, known as the mitotic checkpoint complex [8]. Core spindle checkpoint proteins, such as Mps1, Mad1, Mad2, Bub3 and Mad3/BubR1 assemble or dynamically exchange on unattached kinetochores where at least Mad2 is known to undergo conformational changes essential for optimal binding (and inhibition) of Cdc20 [9,10]. As such the kinetochore seems to function as a catalytic platform upon which a 'wait anaphase' signal is created. Such a catalytic model could be an explanation of how only one single unattached kinetochore can inhibit the APC/C within the entire cell to such an extent that it can delay anaphase onset [11,12].

### The chromosomal passenger complex and the spindle assembly checkpoint: creating unattached kinetochores

**Figure 1:**
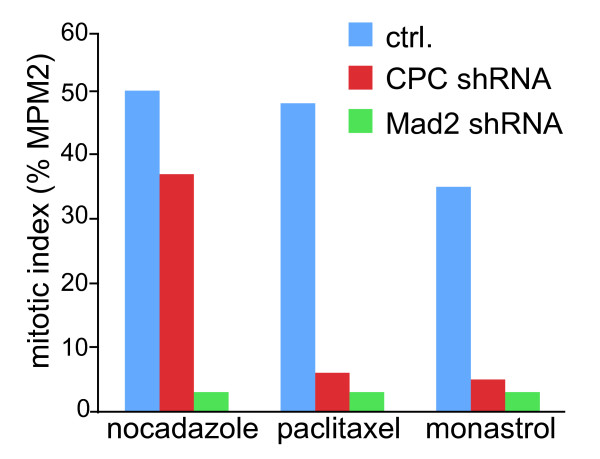
**Different requirements for the chromosomal passenger complex to maintain a mitotic checkpoint arrest induced by different spindle poisons**. Example of an experiment in which osteosarcoma cells (U2OS) were transfected with mock shRNA, Mad2 shRNA, or shRNA for INCENP (which results in knock-down of all chromosomal passenger complex components). Transfected cells were released from a 24 h thymidine block into the indicated drugs. Eighteen hours after the release cells were fixed and prepared for FACS analysis. The MPM2 antibody was used to determine the mitotic index. This type of experiment shows that knock-down of a classical checkpoint protein (Mad2) does not allow cells to accumulate in mitosis with any of the drugs, while knock-down of the chromosomal passenger complex affects the response to paclitaxel and monastrol more dramatically than the response to nocodazole.

Disruption of chromosomal passenger complex function in both yeast and mammalian cells impairs spindle checkpoint activity [13]. Using different spindle poisons to induce a spindle checkpoint-dependent mitotic arrest, it became evident that Aurora B kinase activity was typically required for spindle checkpoint function when microtubules were stabilised by paclitaxel or when monopolar spindles were created through inhibition of Eg5 by monastrol. Yet, a mitotic arrest induced by the microtubule destabilising drug nocodazole was only mildly affected by inhibition of Aurora B kinase activity or by knock-down of chromosomal passenger complex components ([14-17] and figure 1). The major difference between cells treated with these drugs is the presence (paclitaxel and monastrol) or absence (nocodazole) of microtubules that, when present, can bind kinetochores. In monastrol-treated cells, these attachments are mono-oriented and in the case of paclitaxel microtubules have lost their dynamic property and thus have an impaired ability to produce force [18,19]. Therefore these experiments initially led to the idea that, unlike the classical spindle checkpoint proteins, the chromosomal passenger complex was not absolutely required to signal the presence of unattached kinetochores, but only the presence of incorrectly attached kinetochores, *i.e*. kinetochores that are not under tension.

Whereas unattached kinetochores potently promote spindle checkpoint activity and hence APC/C inhibition, it has remained unclear how incorrectly attached kinetochores could signal to the spindle checkpoint. Landmark studies in yeast demonstrated the involvement of Ipl-1, the yeast homolog of Aurora B, in the regulation of kinetochore-microtubule interactions [20] and in promoting chromosome bi-orientation by altering the kinetochore-spindle pole connections [21].

In line with the situation in yeast, inhibition of Aurora B in mammalian cells caused a failure to correct syntelic (kinetochores of sister-chromatids bound by microtubules from the same spindle pole) and merotelic (one kinetochore bound by microtubules from opposing poles) attachments [15,22-24]. Two important kinetochore-localised microtubule-capture factors, the Hec1/Ndc80 and Dam1 complexes, were shown to be Aurora B/Ipl-1 substrates that provide a rationale for how this kinase promotes kinetochore-microtubule error-correction [25]. Phosphorylation of Hec1/Ndc80 reduces its affinity for microtubules *in vitro *and mutation of the putative Aurora B phosphorylation sites stabilises kinetochore-microtubule interactions *in vivo *[26,27].

**Figure 2:**
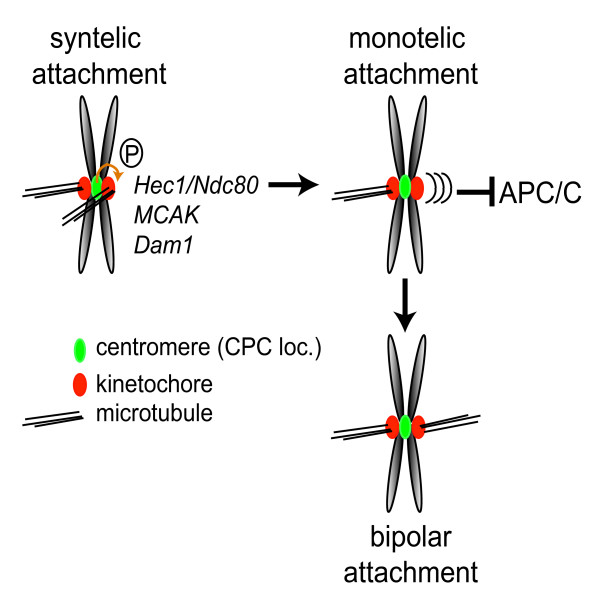
**The chromosomal passenger complex influences spindle checkpoint activity indirectly, through the creation of unattached kinetochores**. Upon entry into mitosis chromosomes start to make random connections with microtubules from the mitotic spindle. To finally obtain bipolar spindle attachments, improper attachments (syntelic – depicted here-, and merotelic attachments) need to be corrected to prevent chromosome segregation errors. Aurora B in complex with its fellow passenger proteins is necessary for this correction process. Through the phosphorylation of kinetochore proteins that bind microtubules it modifies the stability or affinity of the kinetochore-microtubule interaction. As a consequence microtubules detach from the kinetochore allowing new rounds of attachments until bipolarity is obtained. However, during this correction process unattached are created capable of inhibiting the APC/C [33]. As such Aurora B's role in checkpoint function can be considered an indirect consequence of its microtubule destabilising activity.

Additionally, several subunits of the budding yeast Dam1-ring complex are substrates of Ipl-1, and preventing Ipl-1 phosphorylation of this complex caused chromosome segregation defects similar to those found in Ipl-1 mutants [25]. It should be noted that so far no homologues of the Dam1-complex have been found in organisms outside budding and fission yeast. Yet, several studies did identify novel kinetochore and spindle-localised proteins in vertebrates (*i.e*. Ska1/2 and Cep57) that seem to share some functional characteristics with the yeast Dam1-complex [28-30]. It will be important to determine whether these proteins are Aurora B substrates and if phosphorylation by Aurora B changes their functional properties. Besides the Hec1/Ndc80 and Dam1 complexes, Aurora B also influences the localisation and function of the centromere-associated kinesin, MCAK [31,32], and this regulation has been suggested to be of particular importance for the correction of merotelic attachments [24]. Collectively, these findings have resulted in the attractive concept that the main function of Aurora B in complex with its fellow passenger proteins, is to sense and correct faulty attached kinetochore-microtubule interactions. By severing these incorrect attachments it allows new rounds of attachments until bipolarity is obtained. Obviously, during this Aurora B-dependent correction procedure unattached kinetochores are temporarily generated, capable of inhibiting the APC/C. As such, this concept implicates that Aurora B's involvement in spindle checkpoint function is an indirect consequence of its kinetochore-microtubule destabilising activity ([33,34] and figure 2).

### Evidence for a more direct role of the chromosomal passenger complex in spindle checkpoint function

**Figure 3:**
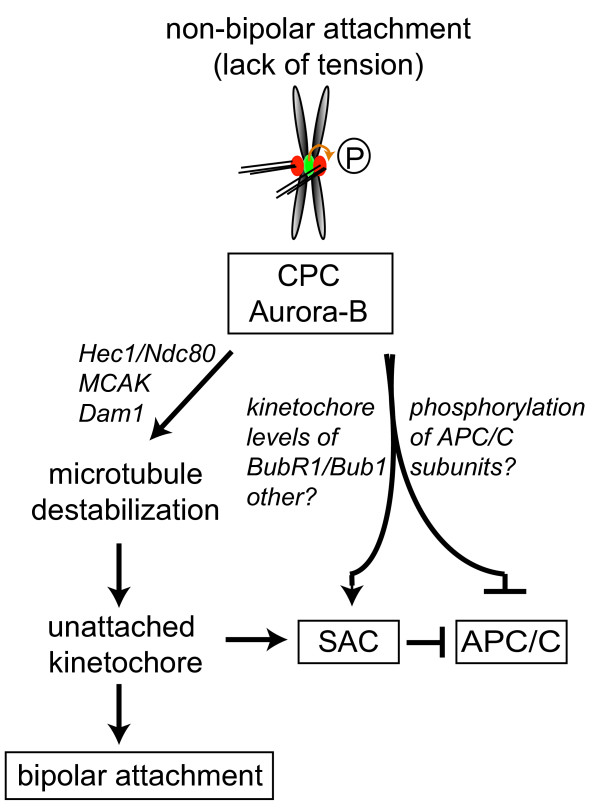
**The chromosomal passenger complex generates an alternative APC/C inhibitory signal that amplifies the 'wait anaphase' signal from unattached kinetochores**. Based on different sets of data (see text for details), we propose that in response to inappropriately attached kinetochores, the chromosomal passenger complex not only destabilises these attachments but also elicits an additional signal that inhibits the APC/C [35]. This could be via direct phosphorylation of APC/C subunits, or via direct control of the spindle assembly checkpoint. Ways be which the chromosomal passenger complex could exert direct control over the spindle checkpoint are through regulation of BubR1/Bub1 kinetochore levels, modulation of the mitotic checkpoint complex or via an as yet unknown pathway. Regardless the mechanism, this additional signal is thought to amplify the unattached kinetochore-derived signal, resulting in a robust checkpoint response when the number of unattached kinetochores is low.

Clearly, a major role for the chromosomal passenger complex in controlling the spindle checkpoint is linked with its ability to generate unattached kinetochores in response to incorrect attachments. However, there are several lines of evidence indicating that the generation of unattached kinetochores is not the sole manner by which the chromosomal passenger complex/Aurora B exerts control over the spindle checkpoint. First, HeLa cells blocked in mitosis by nocodazole (a situation where none of the kinetochores are attached and the microtubule destabilising activity of Aurora B is not relevant) do eventually exit mitosis prematurely after chemical Aurora B inhibition [15]. Second, also RNAi mediated-depletion of chromosomal passenger complex components influences the robustness of the mitotic arrest of nocodazole treated cells [14,16,35] and figure 1). Third, Aurora B becomes critical for a nocodazole-induced mitotic checkpoint arrest in the absence of Bub1 [36], suggesting it might collaborate with other kinases to enforce the mitotic arrest.  Fourth, experiments in Xenopus tissue culture cells as well as studies in fission yeast have shown a requirement for Aurora B activity in spindle checkpoint function in response to unattached kinetochores [37,38]. And fifth, when Aurora B is in a complex with an INCENP molecule that lacks its coiled-coil domain, Aurora B's microtubule destabilising function and hence its capacity to create unattached kinetochores is still intact, yet it is incapable of supporting checkpoint function [35].  This defect became particularly apparent when cells were treated with the microtubule-stabilising drug paclitaxel. Normally, paclitaxel treatment results in a mitotic arrest that is characterised by the lack of tension on all sister chromatids [19] and by the presence of a limited number of unattached kinetochores that are created under the influence of the chromosomal passenger complex [35]. In cells expressing a chromosomal passenger complex lacking the coiled-coil domain of INCENP, unattached kinetochores (that recruit spindle assembly checkpoint proteins like Mad1 and Mad2) could be created in paclitaxel, but the mitotic checkpoint arrest in paclitaxel was completely abrogated [35]. Together, these findings provide strong evidence that the chromosomal passenger complex influences spindle checkpoint function also in a more direct fashion, independent of its well-established destabilising effect on microtubule-kinetochore interactions. It is compelling to speculate that the chromosomal passenger complex is somehow also capable of generating an APC/C inhibitory signal that can amplify or at least work in conjunction with the 'unattached kinetochore-derived' checkpoint signal (figure 3).

### Potential downstream targets of the chromosomal passenger complex involved in spindle assembly checkpoint control

There are several reasons to consider the checkpoint proteins Bub1 and BubR1 as targets of the chromosomal passenger complex. For one, the kinetochore localisation of both proteins requires Aurora B activity [14-17], but it is at present unclear how important kinetochore-recruitment of Bub1/BubR1 is for proper checkpoint function, and how Aurora B controls localisation of BubR1 and Bub1. Moreover, besides functioning in the checkpoint, BubR1 and Bub1 have other duties at the kinetochore. Both proteins are required for kinetochore-microtubule attachments [14,39,40] and Bub1 also controls sister chromatid cohesion [41,42]. Therefore, Aurora B-dependent kinetochore localisation of BubR1/Bub1 could be a reflection of these functions rather than of their function in the spindle checkpoint.

In budding yeast, phosphorylation of Mad3 (the BubR1 orthologue) by Ipl-1 plays a role in checkpoint control [43]. However, we and others have failed to find evidence for direct phosphorylation of BubR1 by Aurora B in human cells ([44] and our unpublished observations). Therefore, it seems likely that BubR1 function is controlled by Aurora B in a more complex fashion in metazoans. Still, the mitotic phosphorylation-status of BubR1 in human cells does depend on Aurora B kinase activity [14]. Interestingly, BubR1 was recently shown to be a substrate of Plk1 and phosphorylation by this kinase is responsible for the phosphorylation-dependent mobility shift of BubR1. This phosphorylation is thought to occur locally when both Plk1 and BubR1 are localised on the kinetochore [44]. It is thus possible that Aurora B merely controls kinetochore localisation of BubR1, and as such indirectly promotes BubR1 phosphorylation by Plk1 (and potentially other kinases). The reduced BubR1 phosphorylation in Aurora B-deficient cells could therefore be a reflection of BubR1 mislocalisation. In this respect, the recent finding that BubR1 (and Bub1) kinetochore recruitment depends on their interaction with Blinkin/KNL-1, a component of the KMN (KNL-1/Mis12/Ndc80/Hec1) protein complex [45], is interesting, since Aurora B influences the (microtubule-binding) function of this complex through direct phosphorylation of Ndc80/Hec1 [26,27]. One could thus speculate that Aurora B controls both microtubule binding and BubR1/Bub1 recruitment by modulating the KMN-complex at incorrectly attached kinetochores. Taken together, it is not yet clear what the downstream targets of the chromosomal passenger complex/Aurora B are in exerting direct control over the spindle assembly checkpoint. Although, Bub1 and BubR1 are attractive candidates, the chromosomal passenger complex (and Aurora B) might very well control spindle checkpoint function through direct modification of other spindle checkpoint components or APC/C subunits.

### Attachment and tension

When correctly attached, kinetochores of sister-chromatids are bound by microtubules from the opposing spindle poles (bipolar attachment) that pull in opposite directions to create physical force (tension) between the still cohered sister-kinetochores [46]. It is a matter of debate whether the mere lack of tension (thus without the presence of unattached kinetochores) is capable of maintaining spindle checkpoint activity (*i.e*. whether there exists a 'tension-only'-checkpoint) and if the chromosomal passenger complex is involved in this. Classical micromanipulation experiments in grasshopper spermatocytes demonstrated that application of tension to an improperly attached chromosome silenced the spindle assembly checkpoint [47]. In addition, experiments in budding yeast showed that the presence of unreplicated chromatids (that cannot generate tension because they lack a sister chromatid) caused a spindle checkpoint-dependent arrest in mitosis [48,49]. These studies indicated the presence of a tension-specific checkpoint branch, but an inherent problem interpreting these findings is that microtubule-kinetochore attachments that do not generate tension are intrinsically unstable [46,50], presumably due to the activity of Aurora B. These unstable kinetochore-microtubule interactions may thus frequently result in the generation of unattached kinetochores. Because of this inherent lack-of-tension consequence, it is very difficult to unequivocally prove the presence of a tension-specific checkpoint signal capable of arresting the cell cycle in the complete absence of unattached kinetochores. Another study in which mammalian Ptk1 cells were cultured at low temperatures showed that these cells were delayed in metaphase [51]. In these metaphase-delayed cells, all kinetochores had acquired normal numbers of kinetochore microtubules [52] but tension between sister-kinetochores was reduced, providing the strongest evidence so far for presence of a 'tension-only'-checkpoint in mammalian cells. Nonetheless, the question whether the absence of tension can function as a direct regulator of the spindle assembly checkpoint is a very important conceptual one that deserves additional investigation. To further test whether solely the lack of tension can inhibit the APC/C, a situation should be created in which tension-lacking microtubule-kinetochore interactions exist that cannot be destabilised by Aurora B. A possible approach to achieve this would be to prevent phosphorylation of microtubule-capture and destabilising factors (*i.e*. Ndc80/Hec1, Dam1 and MCAK) that are controlled by Aurora B [25-27,31,32], thereby artificially stabilising (defective) attachments. Upon combination of such mutations with a situation that prevents the build-up of tension (*e.g*. by using cohesin or shugoshin mutants or by generating single individual chromatids in replication defective (Cdc6) mutants or by paclitaxel/monastrol treatment) [48,53-56], such an experimental setup should be capable of putting to the test the presence or absence of a direct tension-specific checkpoint branch. In such experiments, rigorous tests should be performed that are able to detect the presence or absence of (transient) unattached kinetochores. Obviously, generating such an experimental setup in human cells is a technically challenging endeavour, also because it is likely that not all Aurora B substrates influencing kinetochore-microtubule interactions have been identified. However, since antibody-induced stabilisation of the Ndc80/Hec1-microtubule interaction was shown to generate extremely stable microtubule-kinetochore interactions in the presence of Aurora B activity [27], merely mutating Aurora B phosphorylations on Ndc80/Hec1 might suffice to prevent the creation of unattachments in response to a lack of tension. Additionally, it will be important to understand the mechanisms behind BubR1 kinetochore recruitment in response to lack of tension [51,57] and to assess if (similar to what occurs with Mad2 on unattached kinetochores) BubR1 is modified specifically on these kinetochores to function as a potential APC/C inhibitor in the cytoplasm. Combined, such studies should provide more insight into the potential presence and molecular workings of tension-specific checkpoint signals and their dependency on Aurora B activity.

### Conclusions: A model for spindle checkpoint control by the chromosomal passenger complex/Aurora B

In general, the time cells remain arrested in mitosis upon treatment with spindle poisons greatly varies between cell types, yet eventually all cells do exit from mitosis in the presence of improperly attached or unattached kinetochores. This event is referred to as mitotic 'slippage' and coincides with gradual (proteasome-dependent) destruction of cyclin B [58]. Once the level of cyclin B has dropped below a certain critical treshold, cells exit from mitosis even when all kinetochores are unattached and able to recruit Mad2. These findings were explained by the idea that even a fully functional spindle assembly checkpoint is not capable of blocking all APC/C's from ubiquitinating cyclin B [58]. In line with this reasoning, we propose that Aurora B may influence the efficiency of APC/C inhibition independent from its microtubule-destabilising activity, either by influencing the spindle assembly checkpoint (*e.g*. via affecting the kinetochore levels of BubR1/Bub1 or by regulating mitotic checkpoint complex-dependent inhibition of the APC/C), or by direct phosphorylation of APC/C components (figure 3). The net result would be a more rapid degradation of cyclin B in mitotic cells devoid of active Aurora B. This would explain why the mitotic residence time in nocodazole is shortened after inhibition of Aurora B, and why cells exit more quickly upon Aurora B inhibition after a prolonged mitotic arrest [14,15].

**Figure 4:**
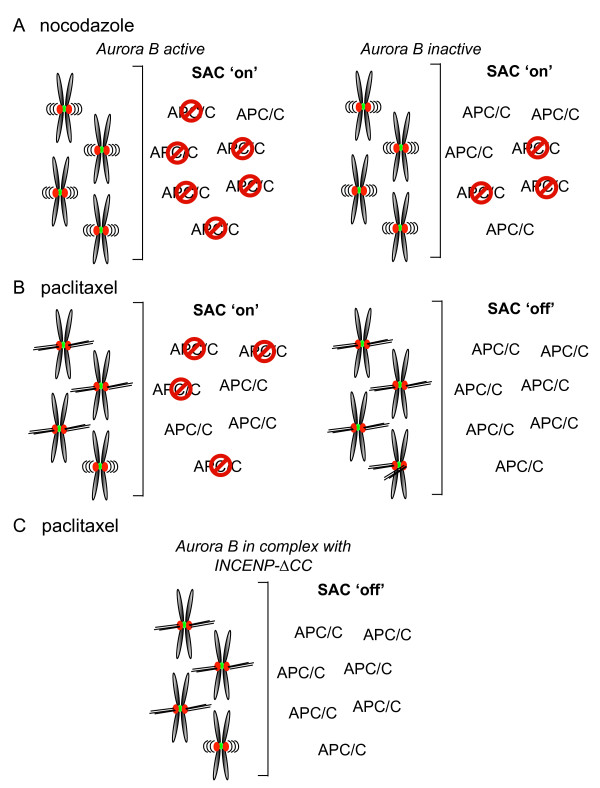
**Model explaining the role of the chromosomal passenger complex/Aurora B in spindle checkpoint function**. (A) Treatment with the microtubule destabilising drug nocodazole results in a long checkpoint-dependent mitotic arrest. However, even when all kinetochores are unattached the spindle assembly checkpoint is not capable of inhibiting all APC/C's which might explain why these cells do eventually exit from mitosis (mitotic slippage) [58]. If Aurora B is inactivated in these cells, less APC/C's will be inhibited. Still this is sufficient to allow a mitotic delay, but this delay is significantly shorter than when Aurora B is active [14, 15]. (B) Treatment with the microtubule stabilising agent paclitaxel induces a mitotic arrest with a few unattached kinetochores [35] most likely inhibiting less APC/C's than when all kinetochores are unattached. Yet, this number of inhibited APC/C's is sufficient to sustain a robust checkpoint-dependent arrest. Since the unattached kinetochores are generated under the influence of the chromosomal passenger complex/Aurora B [35], inhibition of Aurora B will now silence both the unattached kinetochore-derived checkpoint signal and the additional amplification signal, resulting in an override of the spindle assembly checkpoint. (C) Expression of a chromosomal passenger complex that lacks the coiled-coil domain of INCENP does not affect the microtubule destabilising activity of Aurora B. In the presence of paclitaxel unattached kinetochores are therefore generated but this does not result in a checkpoint-dependent arrest [35]. We propose that due to the low number of unattached kinetochores that are now inhibiting the APC/C, the spindle checkpoint becomes more dependent on this additional chromosomal passenger complex-generated amplification signal to inhibit a sufficient number of APC/C's that allow a robust mitotic arrest.

Along the same lines, if even in the presence of 100% unattached kinetochores the spindle checkpoint is not capable of fully inhibiting all APC/C's from ubiquitinating cyclin B, a few or a single unattached kinetochore may be able to block even fewer APC/C's, resulting in a more rapid degradation of cyclin B and hence a less robust checkpoint arrest. If now the efficiency of APC/C inhibition is further crippled by defects in chromosomal passenger complex/Aurora B function (*e.g*. the situation in which Aurora B is in complex with INCENP-ΔCC), cells will swiftly exit mitosis even in the presence of a few unattached kinetochores [35]. Clearly, this additional function of Aurora B in checkpoint control is only revealed when its microtubule destabilising function is unperturbed (*i.e*. when in complex with INCENP-ΔCC) or not relevant (*i.e*. in nocodazole), and is expected to become more critical when only a few kinetochores are unattached (*i.e*. INCENP-ΔCC expressing cells treated with paclitaxel) (figure 4). Thus, we propose that when sister-kinetochores are improperly attached (and hence fail to generate tension), the chromosomal passenger complex/Aurora B response to this defect is twofold: (i) by severing the kinetochore-microtubule attachments, it will create unattached kinetochores capable of inhibiting the APC/C, (ii) by eliciting an additional APC/C inhibitory signal, it will amplify/strengthen the unattached kinetochore-derived signal thereby ensuring a robust spindle checkpoint response when the number of unattached kinetochores is low.

### Future directions

Recently, Aurora B substrates have been identified (*e.g*. Hec1/Ndc80, MCAK, Dam1 [25-27,31,32]) involved in the regulation of kinetochore-microtubule interactions. Surely, many more substrates involved in kinetochore-microtubule stability await identification, and an additional challenge will be to find the Aurora B substrates that will give mechanistic insight into how the chromosomal passenger complex exerts direct control over the spindle checkpoint. Not only the identity of the Aurora B substrates are gradually revealed, also its mode of activation appears to be more complicated than merely through the interaction with the IN-box of INCENP [59].

Recent studies in Xenopus extracts showed that microtubules and the passenger protein TD60 are required for optimal activation of Aurora B and that certain substrates first need to be phosphorylated by other kinases, (*e.g*. Plk1 and Haspin) to relieve their inhibitory effect on Aurora B activity [60]. In addition, phosphorylation of borealin by Mps1 also contributes to the activation of Aurora B [61]. Taken together, this suggests that also the interplay between the chromosomal passenger complex and certain kinases could result in a mode of Aurora B activation that may define its substrate specificity and function.

In figure 2 we have drawn the scenario in which Aurora B specifically destabilises the incorrect kinetochore-microtubule interaction. However, it is still not known if Aurora B is in fact able to discriminate between these two types of attached kinetochores and if so, how the chromosomal passenger complex/Aurora B is able to sense these faulty attachments that fail to generate tension. Recently, the coiled-coil domain within INCENP has been suggested to function as the tension-sensor within the chromosomal passenger complex through its interaction with microtubules [62], but it is still largely unknown how the INCENP-microtubule interaction could translate into an increase in Aurora B activity. Alternatively, it may very well be that in prometaphase Aurora B creates a general state of loose or dynamic kinetochore-microtubule attachments as a consequence of Hec1/Ndc80 phosphorylation and MCAK regulation [26,27,63] and that upon obtaining bipolar attachments and hence tension, Aurora B activity is constrained as a consequence of spatial separation from its kinetochore substrates [21,64], resulting in stabilisation of the correct attachments. Finally, understanding the mechanisms behind the spindle checkpoint-associated function of the chromosomal passenger complex might yield new insights into the development of chromosomal instability in cancer cells.

## Authors' contributions

GV drafted the manuscript, AFM contributed to the conception of the model, SMAL drafted and wrote the manuscript. All authors read and approved the final manuscript.
